# Vocabulary Repetition Following Multisensory Instruction Is Ineffective on L2 Sentence Comprehension: Evidence From the N400

**DOI:** 10.3389/fpsyg.2022.707234

**Published:** 2022-01-28

**Authors:** Reza Pishghadam, Haniyeh Jajarmi, Shaghayegh Shayesteh, Azin Khodaverdi, Hossein Nassaji

**Affiliations:** ^1^Department of English, Ferdowsi University of Mashhad, Mashhad, Iran; ^2^Department of English, Bahar Institute of Higher Education, Mashhad, Iran; ^3^Department of English, University of Victoria, Victoria, BC, Canada

**Keywords:** multisensory instruction, emotioncy, event-related brain potentials (ERPs), N400, repetition

## Abstract

Putting the principles of multisensory teaching into practice, this study investigated the effect of audio-visual vocabulary repetition on L2 sentence comprehension. Forty participants were randomly assigned to experimental and control groups. A sensory-based model of instruction (i.e., emotioncy) was used to teach a list of unfamiliar vocabularies to the two groups. Following the instruction, the experimental group repeated the instructed words twice, while the control group received no vocabulary repetition. Afterward, their electrophysiological neural activities were recorded through electroencephalography while doing a sentence acceptability judgment task with 216 sentences under acceptable (correct) and unacceptable (pragmatically violated) conditions. A one-way analysis of variance (ANOVA), a multivariate analysis of variance (MANOVA), and a Bayesian repeated-measures ANOVA were used to compare the behavioral and neurocognitive responses [N400 as the main language-related event-related brain potential (ERP) effect] of the two groups. The results showed no significant N400 amplitude difference in favor of any of the groups. The findings corroborated the ineffectiveness of two repetitions preceded by multisensory instruction on L2 sentence comprehension.

## Introduction

Comprehension in general and sentence comprehension, in particular, have been the pinnacle of many cognitive studies on L2 (e.g., Newman et al., 2012; Zheng and Lemhöfer, 2019). In such studies, sentence processing has been examined from multiple perspectives, including syntactic (Embick et al., 2000) and semantic (Dapretto and Bookheimer, 1999; Bookheimer, 2002). The semantic processing of a sentence relies, to a large extent, on the processing of individual words of that sentence. Therefore, vocabulary retention plays a significant role, hastening or hindering this process.

To improve vocabulary learning and retention, different strategies have been employed. As for one, central to vocabulary learning as a gradual process is the concept of repetition (Nation, 2015). Repetition is known to be mechanical or meaningful depending on the teaching methodology applied by teachers. Along with the changes in language teaching methodology from audio-lingual classroom drills grounded in the theory of behaviorism to communicative approaches, vocabulary repetition was constantly shaped and reshaped. Meaningful repetition practices took priority over simple mechanical ones to make learning more enduring (Horst, 2013; Kartchava and Nassaji, 2019; Hidalgo and Garcia Mayo, 2021).

Although researchers jointly agree that learning depends on the degree of any type of repetition with more repetitions leading to the better learning of the points (e.g., Thalheimer, 2003; Chen and Truscott, 2010; Laufer and Rozovski-Roitblat, 2011), there has been considerable debate over the optimal number of repetitions that ensures vocabulary learning and boosts comprehension (Peters, 2014; Nation, 2015; Liu, 2018). While Horst et al. (1998); Waring and Takaki (2003), and Webb (2007) respectively found 8, 10, and 12 repetitions as the optimal number, Vidal (2011) minimized the frequency to two and three meaningful repetitions in a reading context.

To capture the repetition effect, different approaches have been adopted. Unlike conventional approaches, which basically target learners’ performance and achievement, most recent studies have endeavored to employ neurocognitive tasks to get more reliable results. By virtue of this objective inspection, a few studies documented that repetition may have no effect (e.g., Amir Kassim et al., 2018) or even a negative effect (e.g., Peterson and Mulligan, 2012; Mulligan and Peterson, 2013) on human memory. As for one, Amir Kassim et al. (2018) deduced that, unlike visual and a combination of auditory and visual repetitions, two auditory repetitions have no effect on the participants’ recognition memory. Not only that, Peterson and Mulligan (2012) reported a negative repetition effect for the participants who went through a list of cue-target pairs twice compared to those who studied the pairs once only.

To further substantiate the findings, neurolinguists set out to record and examine the brain activity of the learners through electroencephalography (EEG) and event-related brain potential (ERP) techniques, evidencing that the human brain responds emphatically to any type of repetition (Van Strien et al., 2007). A review of the related literature reveals that such neurophysiological studies have mainly investigated the word repetition effect on the basis of pertinent ERP component modulations during the process of repetition (Henson, 2003; Maccotta and Buckner, 2004; Van Strien et al., 2007). Yet, the missing chain in the literature is how these word repetitions affect the overall comprehension of the learners.

To delve into the electrophysiological underpinning of sentence comprehension, researchers (e.g., Hagoort et al., 2004; Hald et al., 2006; Kos et al., 2012) have designed different sentence acceptability judgment tasks with semantically violated (sentences with the word knowledge violation, e.g., a caper is kind.) and pragmatically violated sentences (sentences with the world knowledge violation, e.g., a caper is sweet.). According to their findings, the neurocognitive mechanism of sentence comprehension manifests itself in a series of ERP components, with N400 as the most general component, providing insights into the neurobiology of meaning. The N400 effect, with its peak around 400 ms following the stimulus, is sensitive to semantic modifications (Xu et al., 2015; Payne et al., 2019). The amplitude of this negative-going deflection is basically defined by the degree of congruence between a word and its sentential context and the load of cognitive endeavor required to access the semantic memory (Kutas and Federmeier, 2000). The component is similarly influenced by other variables such as verbal working memory (e.g., Brown and Hagoort, 1999), word frequency (Dambacher et al., 2006), presentation modality (Kutas and Federmeier, 2011), and word priming (McRae et al., 2005). These amplitude changes may bring about different degrees of comprehension.

For improved comprehension, which is closely linked to vocabulary retention, not only vocabulary repetition practices but the nature of instruction may serve a pivotal role. It is commonly believed that a rudimentary path to deep processing and enhanced learning is the involvement of the senses. Underpinning the importance of senses in learning a language (Massaro, 2004), sensory teaching, pioneered by Montessori (1912), has been used by educators and teachers believing that senses, either in isolation or in different combinations, give way to inclusive learning, which engages all the learners with different needs (Hockings, 2010; Katai, 2011). Brain research findings have similarly corroborated the effect of multisensory instruction (MSI) on brain performance, particularly sentence comprehension, which is improved by the involvement of more sensory information channels and neural structures as a result of the interaction of more senses (Shams and Seitz, 2008; Pishghadam et al., 2020, 2021; Shayesteh et al., 2020).

**FIGURE 1:**
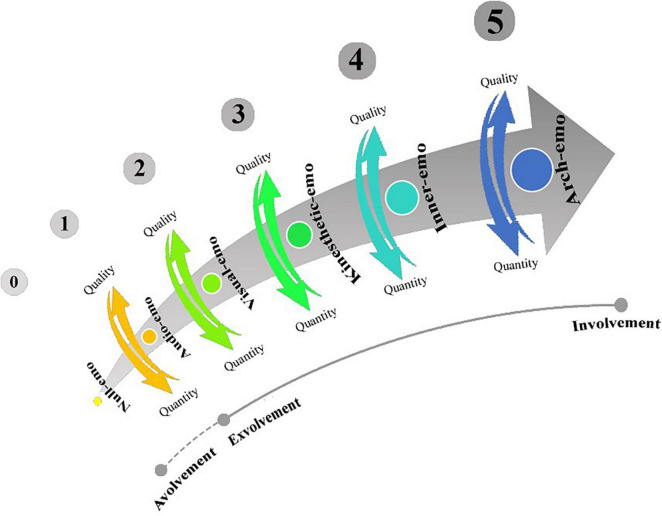
Emotioncy levels [reprinted with permission from “Emotioncy, extraversion, and anxiety in willingness to communicate in English,” by Pishghadam (2016), Proceedings of the 5th International Conference on Language, Education, and Innovation. London, United Kingdom].

What we hypothesize in this study is that, given the effectiveness of MSI in engendering in-depth learning (Baines, 2008), vocabulary repetition is likely to be redundant for sentence comprehension. To verify that, we used a validated sensory-based model of instruction, coined as *emotioncy* (a blend of emotion + frequency), and combined the five senses of auditory, visual, tactile, olfactory, and gustatory (see Pishghadam et al., 2017, 2020, 2021; Karami et al., 2019; Makiabadi et al., 2019; Shayesteh et al., 2020; Boustani et al., 2021). The model (Figure 1) presents us with two major combinations of the senses, namely *exvolvement* (i.e., a combination of auditory, visual, and tactile/kinesthetic) and *involvement* (i.e., a combination of auditory, visual, and tactile/kinesthetic, olfactory, and gustatory).

To decide upon the number of vocabulary repetitions following our MSI, we drew upon the findings of a recent, relevant study conducted by Jajarmi et al. (2020). They adopted a bisensory (auditory + visual) approach according to the emotioncy model and taught a list of unknown English vocabulary items to a group of language learners. They used different numbers of repetitions to eventually come up with the minimum number of effective repetitions. Quite in line with Hintzman (1970), Nelson (1977), and Phaf (2012), using paper and pencil tests, Jajarmi et al. (2020) reported two repetitions as the threshold for making significant changes in vocabulary learning and that, one, three, four, and five repetitions make no further contribution to vocabulary learning as a result of bisensory instruction.

For the purpose of this study, and to examine if sentence comprehension is influenced by two vocabulary repetitions (as the minimum number of effective repetitions) following the MSI, we selected six vocabulary items of which the learners had no previous knowledge. We taught the words to a control group and an experimental group of participants. The control group received the MSI only, whereas the experimental group had the MSI followed by two audio-visual repetitions of the vocabulary items (see section “The Instruction” for details on the procedure). Thereafter, we compared the immediate behavioral and cognitive performance of the two groups, on a sentence acceptability judgment task (with correct and pragmatically violated sentences), for any probable neural response differences associated with sentence comprehension. In order to evaluate the differences, the ERP technique was employed. Based on the previous studies acknowledging the efficiency of using multiple senses in the process of learning and comprehension, and the ERP studies recognizing the N400 as an indicator of semantic access difficulty, we predicated that two vocabulary repetitions following the MSI may not reduce the N400 amplitude and facilitate semantic access during sentence comprehension. Therefore, we expect to observe no N400 amplitude difference between the control and experimental groups, concluding that MSI is a working theory that is not influenced by two vocabulary repetitions.

## Materials and Methods

### Participants

Forty-five (33 female and 12 male) native speakers of Persian, with English as their foreign language, volunteered to take our pretests, 3 of whom were not recruited to participate in the ERP experiment due to their pretest results. Moreover, the data for two of the participants were discarded because of excessive eye movement and muscle artifact. The participants’ age ranged from 18 to 30 years (*M* = 21.7, *SD* = 2.6). They were all right-handed (Oldfield, 1971), neurologically healthy, and had normal or corrected-to-normal vision. All of them were at the intermediate level of language proficiency, and their working memory score ranged from 10 to 12 (*M* = 11.3, *SD* = 1.3) (Wechsler, 1981). They neither had participated in the pilot tests nor had any knowledge of the six selected vocabulary items they were supposed to learn. For the purpose of this study, the participants were randomly assigned to a control group (G1, *N* = 20) and an experimental group (G2, *N* = 20). The participants gave written informed consent under a protocol approved by the Ferdowsi University of Mashhad Ethics Committee before the experiment and took part in the research according to their willingness to participate. They received either course credits or gifts for their participation.

### Materials

#### Pretest Materials

##### The Emotioncy Scale

In order to make sure that the participants had no knowledge of the selected items for the experiment, an emotioncy scale was used (Borsipour, 2016). Each item measured the participants’ familiarity with the target words through a 6-point Likert scale with (1) not familiar; (2) heard; (3) heard and seen; (4) heard, seen, and touched; (5) heard, seen, touched, and used; and (6) heard, seen, touched, used, and done research on. The participants who had prior experiences with any of the six words were excluded in this phase.

##### The Oxford Quick Placement Test

The Oxford Quick Placement Test (OQPT) (Allan, 1992) was administered to measure the participants’ English proficiency level. This test has two parts, each containing 40 and 20 items, respectively. The items are in multiple-choice and cloze test formats, and the time to respond to the questions is 30 min. In this test, the obtained scores of 30–40 represent intermediate proficiency level in English.

##### The Digit Span Subtest of the Wechsler Adult Intelligence Scale III

Since working memory and specifically the phonological loop plays a substantial role in vocabulary learning and vocabulary retention (Gillam, 2002), we used the digit span subtest of the Wechsler Adult Intelligence Scale III (WAIS-III) (Wechsler, 1981) as a measure of homogeneity. Given that based on the results, the mean span for those who took the test was 11 with a standard deviation of 1, we selected those participants within the limited range of 10–12.

##### The Edinburgh Inventory of Handedness

The Edinburgh inventory of handedness (Oldfield, 1971), as a measure of hand laterality, was used to select right-handed individuals. The inventory includes 10 questions about writing, drawing, throwing, using scissors, using a toothbrush, using a knife (without a fork), using a spoon, using a broom (upper hand), striking a match, and opening a box, along with two supplementary questions: “which foot do you prefer to kick with?” and “which eye do you use when using only one?” According to the scale, the participants who did more than two of the mentioned activities with their left hand were excluded from the study.

#### Stimulus Materials (Vocabulary Items)

**FIGURE 2:**
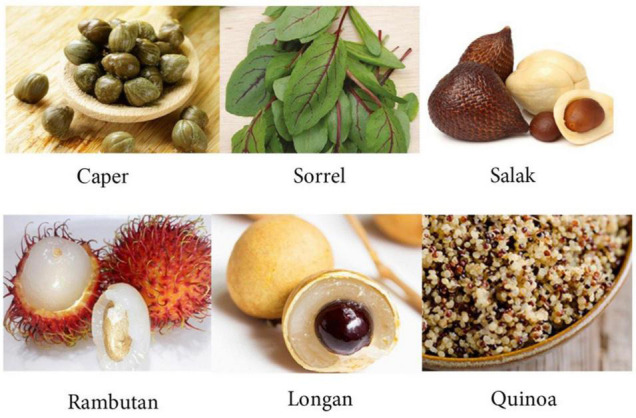
The six selected vocabulary items. Images reproduced with permission from www.pinterest.com.

In order to choose the six vocabulary items, a list of 48 words (along with their Persian translation) which were the names of some edible things, including fruits, plants, and vegetables, was culled and put into the emotioncy scale (Borsipour, 2016). It should be mentioned that the translations of the words were not cognates in the L1 of the participants. Then the scale was randomly administered to 150 respondents (87 females and 63 males), who were different from the main participants of the study. Finally, six words of which 95% of the respondents had no prior knowledge, were selected for the MSI. The words were *caper, longan, sorrel, salak, rambutan*, and *quinoa* (Figure 2).

### Procedure

#### The Sentence Acceptability Judgment Task

A sentence acceptability judgment task was constructed according to a framework presented by Hagoort et al. (2004), Hald et al. (2006), and Kos et al. (2012), using Psychophysics Toolbox Version 3 (PTB-3) for MATLAB (version 2015a, The MathWorks, MA, United States). The task required the participants to judge the acceptability of the sentences they saw word by word on the screen. The six instructed vocabulary items were embedded in a number of 144 sentences (72 sentence pairs with 3–8 words each). Each pair comprised acceptable and unacceptable conditions of a sentence: a non-violated correct sentence (Co) and a sentence with pragmatic violation (Pr). In order to avoid conditioning, 72 unrelated sentences of similar length, complexity, and structure (36 correct and 36 pragmatically violated), including the names of animals, fruits, and objects, were added as fillers, making 216 sentences in total (Table 1). Each sentence pair was identical except for one word only (i.e., the critical word), which appeared at the sentence-final position. The critical words were matched across the two conditions in terms of average length in characters, word class, bigram frequency, and cloze probability (checked by two native speakers).

**TABLE 1:** Example Sentences of the two different conditions in the sentence pairs in addition to the filler sentences.

Sentence type	Condition	Example sentences
Target	Correct	A **salak** looks like a *fig*.
	Pragmatically violated	A **salak** looks like a *cherry*.
Filler	Correct	A monkey has a tail.
	Pragmatically violated	A monkey has a horn.

*The critical words are in italics. The target words are boldfaced.*

**FIGURE 3:**
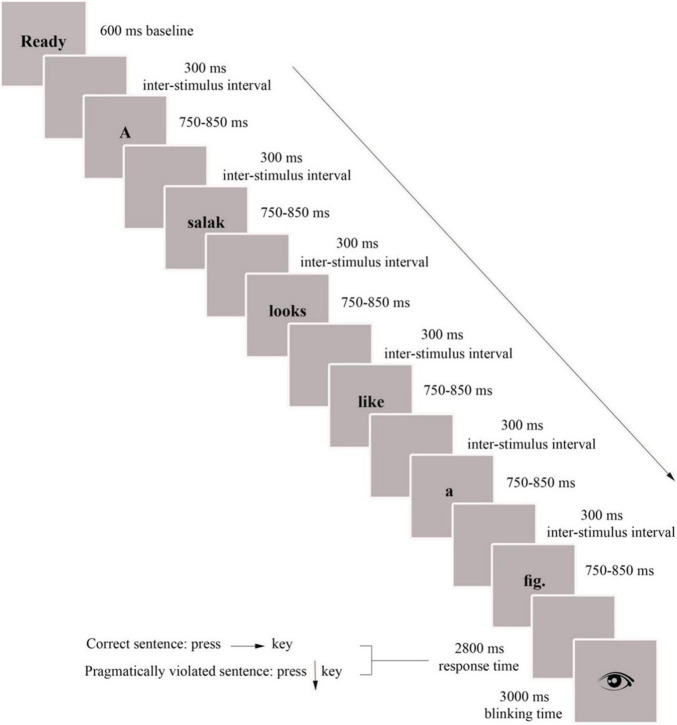
Screen simulations and temporal sequence of an experimental block of the sentence acceptability judgment task.

The task consisted of three 10-min experimental blocks separated by two short breaks. In each block, each sentence was presented word by word in the center of the computer screen (Figure 3). The words were boldfaced in black lower case Times New Roman letters with 58-point font size against a light gray background. The first word of each sentence was capitalized, and the final word of each sentence was presented with a period. The viewing distance was about 100 cm for each participant. Each experimental block started with a 600 ms baseline before the stimulus onset. Each word was presented for 750–850 ms (randomly varied to avoid the predictability of the response time (RT) for participants in terms of their reaction times) followed by a blank screen for 300 ms as an inter-stimulus interval (before the appearance of the next word). After the final word, there was a blank screen for 2800 ms in which the participants were supposed to decide on the truthfulness of the sentences by pressing a key. They were asked to press the right arrow key on the computer keyboard if the sentence was correct, the down arrow key if the sentence was pragmatically wrong, and press no key if they did not know the response. After the response window, an eye image was displayed in the center of the screen for 3000 ms, allowing the participants to move their eyes and blink intentionally to prevent eye fatigue. There was a 300-ms blank page between the eye disappearance and the start of the next trial.

#### The Instruction

The data collection was split into pre-experimental (i.e., instruction) and experimental (i.e., ERP recording) phases. During the pre-experimental phase, each participant learned the six vocabulary items through the MSI. For the purpose of the instruction, along with the real fruits and vegetables, a PowerPoint presentation, which contained the name and some different pictures of the items, and a photo booklet were used to make it similar to a classroom environment. The participants received sensory instruction through inner emotioncy, which is the integration of auditory, visual, kinesthetic, smell, and taste, for the six words. The same amount of time was allocated to the instruction of each word. The whole instruction took approximately 20 min. Table 2 presents a sample instruction of one of the words and the information transferred to the participants.

**TABLE 2:** A sample instruction for an involved word (using a combination of auditory, visual, and tactile/kinesthetic, olfactory, and gustatory senses).

Salak (the participants had a salak with a plate and a knife to cut and smell. The instructor was providing the necessary information about the fruit at the same time).	Look at this fruit. This is a Salak. Salak has an alternative name which is “snake fruit.” Guess why. Aha … because … you see… the skin looks like that of a snake, doesn’t it? The skin is very thin but inedible. It is brown like a walnut. You see… the shape is almost like a fig. Tell me about the size… as you see it is as big as a lemon. Now peel it very gently. Try not to hurt the flesh. A salak has three big lobes. The lobes look like garlic. Does the fruit have any seeds? Cut the lobes to find it if any… Aha you see… There is a seed in one of the lobes only. How about the smell? Tasty? Taste it. It is very juicy.

After the instruction, the participants of G1 directly went for the ERP recording, yet the participants of G2 received two audio-visual repetitions for each word. For the first repetition, the participants of G2 were asked to look at the real objects on the desk and repeat the names after the instructor when the instruction was almost over. For the second repetition, they went through the same procedure (i.e., repetition) 15 min later when they had the EEG cap on. There was a time interval of 15 min between the second repetition and the ERP experiment, during which the participants went through a practice block of the task (see section “Electroencephalography Recording”) and got ready for the recording. According to Cepeda et al. (2006), the lag between the repetitions and the retention interval need to approximately match for optimal memory performance.

#### Electroencephalography Recording

The participants were tested individually in a sound-attenuated and dimly illuminated room. Prior to the main experiment, they went through a practice block of 20 items to get acquainted with the task requirements.

The EEG was recorded from 23 active Ag/AgCl-sintered electrodes mounted on an elastic electrode cap (g.GAMMAcap from g.tec medical engineering GmbH). The electrodes were placed according to the 10–20 international system of the American Electroencephalographic Society over midline sites at Fz, FCz, Cz, Pz, and Oz; frontal sites at AF3, AF4, F3, F4, F7, and F8; fronto-central sites at FC3 and FC4; fronto-temporal sites at FT7 and FT8; central sites at C3 and C4; parietal sites at P3, P4, P7, and P8; and occipital sites at PO7 and PO8. The optimal electrode arrangement was determined according to similar studies in the field (e.g., Salmon and Pratt, 2002; Hagoort et al., 2004; Van Berkum et al., 2005; Hald et al., 2006). All electrodes were referenced to the left mastoid and re-referenced to the average of the left and right mastoids. Vertical and horizontal eye movements were monitored *via* three additional electrodes placed above and below the left eye and on the left outer canthus. Electrode impedances were kept below 5 kΩ. The EEG and EOG signals were digitized online with a sampling frequency of 250 Hz and were amplified using the 32-channel wireless g.Nautilus EEG system (gtec, Austria), with a bandpass filter between 0.1 and 70 Hz and a notch filter of 50 Hz.

### Data Analysis

All recorded EEG signal data were imported into MATLAB (MathWorks, 2015b). To analyze the waveforms, MATLAB along with the EEGLAB (an extension of MATLAB software) were used. The EEG data were bandpass filtered between 0.5 and 60 Hz. Afterward, the data were re-referenced to the mean of the linked mastoids. Poor EEG channels were replaced with their interpolated version applied to the remaining channels. No more than 2 channels were interpolated for each participant, with the majority of the interpolated channels positioned at parieto-occipital and occipital sites. High amplitude eye blinks and muscle artifacts were then removed using the Artifact Subspace Reconstruction (ASR) algorithm from the EEGLAB. The remaining high frequencies were eliminated using a low pass filter with a cut-off frequency of 25 Hz. Next, epochs from −200 to 1100 ms, with respect to the onset of the critical word, were segmented to a 200 ms pre-onset baseline. A linear detrend algorithm (using the 200 ms before the stimulus onset to 3 s after) was applied to the epoched data to further remove drifts. Noisy epochs with potentials exceeding ±70 μv were rejected. Finally, all remaining trials (see Table 3 for the descriptive statistics) were averaged.

**TABLE 3:** Descriptive statistics for the number of averaged epochs.

Condition	Group	Min	Max	Mean (out of 72 items)	SD
Correct	G1	49	59	55.68	1.23
	G2	50	57	54.23	2.87
Pragmatically violated	G1	50	60	56.01	0.85
	G2	51	59	56.94	2.21

*Overall there were 72 epochs to average for every condition of each group. Yet, we only averaged the ones to which the participants gave right answer.*

For the critical words of the sentences, N400 was analyzed. Based on the findings of the previous literature (e.g., Danko et al., 2014; Yang et al., 2014; Molinaro et al., 2016; Volz et al., 2019), the N400 component was quantified as the mean amplitude in a latency window of 300–550 ms after the onset of the critical word and relative to a 200 ms baseline.

For the behavioral data analysis, a one-way between-groups analysis of variance (ANOVA) was performed to examine the main effect of repetition on the participants’ acceptability judgment of the correct and pragmatically violated sentences. For the ERP data analysis, as to the main effect of repetition, a multivariate analysis of variance (MANOVA) along with a Bayesian Repeated Measures ANOVA were run to test for the null hypothesis. All the statistical procedures were computed using an alpha level of 0.05, and to reduce the chance of a Type 1 error, Bonferroni adjustment was applied.

## Results

### Behavioral Results

Descriptive statistics of response accuracy (RA) scores and RTs for G1 and G2 are given in Table 4.

**TABLE 4:** Descriptive statistics of response accuracy scores and response times for G1and G2.

	Linguistic condition	Group^a^	Mean (for 72 items)	SD
Response accuracy	Correct	G1	59.05	5.64
		G2	58.85	6.86
	Pragmatically violated	G1	60.80	7.38
		G2	61.95	5.40
	Correct	G1	0.96	0.21
		G2	0.96	0.25
Response time (s)	Pragmatically violated	G1	0.99	0.22
		G2	0.99	0.24

*^a^N = 20.*

A one-way between-groups ANOVA was performed to examine the main effect of repetition on the participants’ acceptability judgment of the correct and pragmatically violated sentences. The results of Levene’s test for homogeneity of variances showed that the assumption of homogeneity of variance was not violated (*p* > 0.05). The difference between the two groups did not reach significance regarding the main effect of repetition on RA in terms of both correct [*F*(1,38) = 0.21, *p* = 0.92, $\eta_{\text{p}}^{2}=0.14$] and pragmatically violated [*F*(1,38) = 0.32, *p* = 0.58, $\eta_{\text{p}}^{2}=0.18$] conditions.

Similarly, the results of RTs failed to show a significant difference between G1 and G2 in the amount of time it took them to judge the truthfulness of the sentences in terms of either of the conditions, that is, correct [*F*(1,38) = 0.33, *p* = 0.97, $\eta_{\text{p}}^{2}=0.21$] and pragmatically violated [*F*(1,38) = 0.19, *p* = 0.89, $\eta_{\text{p}}^{2}=0.19$].

### Event-Related Brain Potential Results

**FIGURE 4:**
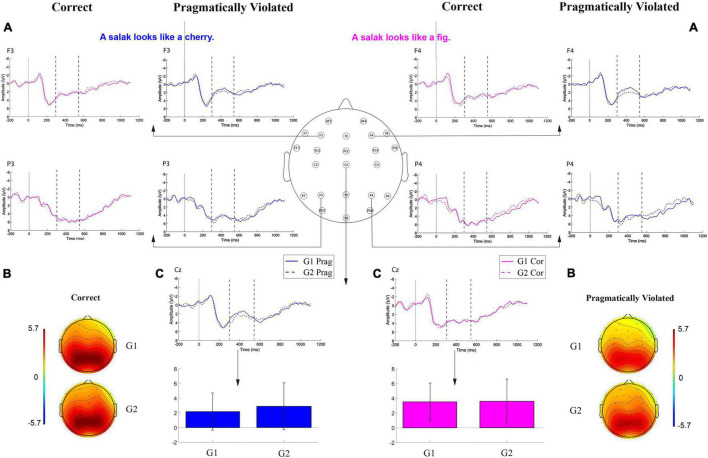
**(A)** Grand-average (*N* = 20 per group) ERPs time-locked to the onset of the critical word for the correct (Cor) and pragmatically violated (Prag) conditions in G1 and G2 at F3/4, P3/4, and Cz as sample locations. **(B)** The topographic maps show the distribution of the N400 for both groups regarding both conditions. **(C)** The bar plots show no significant difference between the two groups with regard to correct (pink) and pragmatically violated sentences (blue).

At the next step, we analyzed the participants’ brain activity, recorded during the task, to find out whether they were in line with the behavioral results. Figure 4 shows grand-average ERPs time-locked to the onset of the critical word for the correct and pragmatically violated conditions in G1 and G2 at F3, F4, P3, P4, and Cz as sample electrode sites from the 23 inspected locations on the scalp. The grand-average waveforms for G1 and G2 showed different neural correlates of sentence processing, including a broadly distributed negative-going deflection (representing the N400) starting at about 300 ms after the onset of the critical word peaking at 400 ms. To find the location of the maximum N400 amplitude, we grouped the 23 electrodes into three regions of interest (ROIs): anterior (AF3/4, F3/4, F7/8, FT7/8, Fz), central (FC3/4, FCz, C3/4, Cz), and posterior (P3/4, P7/8, Pz, PO7/8, Oz). The *F* test result [*F*(2,78) = 17.02, *p* = 0.000; $\eta_{\text{p}}^{2}=0.30$] revealed that the negativity in the anterior areas (*M* = 1.73 μv) was significantly larger than that of the central (*M* = 2.98 μv) and posterior regions (*M* = 3.97 μv).

Consequently, the two groups were compared on the N400 effect at the 23 electrodes. To this end, a one-way between-groups MANOVA was run to investigate group differences in the N400 mean amplitude in terms of the two linguistic conditions (i.e., correct and pragmatically violated). Preliminary assumption testing noted no violations of normality and homogeneity of variance-covariance matrices (*p* > 0.05). The multivariate effect of group regarding the N400 mean amplitudes signaled no significant effect for repetition in terms of the groups [*F*(8, 31) = 0.39, *p* = 0.97; Wilks’ Lambda = 0.40; $\eta_{\text{p}}^{2}=0.60$].

To ensure the lack of difference between the mean amplitudes, the Bayesian Repeated Measures ANOVA [factors: electrode (23), condition (2); between-subject factor: group (G1 & G2)] was used. As Tables 5, 6 show, Bayesian analyses support the null hypothesis, indicating no main or interaction effect of the group (i.e., repetition).

**TABLE 5:** Bayesian repeated measures model comparison for the N400.

Models	P(M)	P(M| data)	BF_M_	BF_10_	Error %
Electrode + condition	0.05	0.70	42.93	1.00	
Electrode + condition + group	0.05	0.17	3.87	0.25	2.48
Electrode + condition + group + condition × group	0.05	0.11	2.41	0.16	3.86
Electrode + condition + group + electrode × group	0.05	2.41e−5	4.34e−4	3.42e−5	2.37
Electrode + condition + group + electrode × group + condition × group	0.05	1.77e−5	3.18e−4	2.51e−5	8.90
Electrode + condition + electrode × condition	0.05	1.19e−5	2.14e−4	1.69e−5	2.31
Electrode + condition + group + electrode × condition	0.05	3.15e−6	5.67e−5	4.47e−6	3.93
Electrode + condition + group + electrode × condition + condition × group	0.05	2.39e−6	4.30e−5	3.39e−6	11.57
Electrode + condition + group + electrode × condition + electrode × group	0.05	4.55e−10	8.19e−9	6.45e−10	3.92
Electrode	0.05	3.98e−10	7.17e−9	5.66e−10	2.03
Electrode + condition + group + electrode × condition + electrode × group + condition × group	0.05	3.16e−10	5.70e−9	4.49e−10	5.04
Electrode + Group	0.05	9.95e−11	1.79e−9	1.41e−10	2.15
Electrode + condition + group + electrode × condition + electrode × group + condition × group + electrode × condition × group	0.05	3.13e−14	5.64e−13	4.45e−14	3.00
Electrode + group + electrode × group	0.05	1.27e−14	2.29e−13	1.80e−14	2.31
Condition	0.05	9.08e−101	1.63e−99	1.28e−100	2.53
Condition + group	0.05	1.97e−101	3.55e−100	2.80e−101	2.31
Condition + group + condition × group	0.05	7.56e−102	1.36e−100	1.07e−101	2.80
Null model (including subject)	0.05	2.52e−107	4.55e−106	3.58e−107	2.01
Group	0.05	5.72e−108	1.03e−106	8.12e−108	2.26

**TABLE 6:** Analysis of effects.

Effects	P(incl)	P(excl)	P(incl| data)	P(excl| data)	BF_incl_
Electrode	0.73	0.26	1.00	0.00	∞
Condition	0.73	0.26	1.00	4.98e−10	7.16e+8
Group	0.73	0.26	0.29	0.70	0.15
Electrode × condition	0.31	0.68	1.74e−5	1.00	3.78e−5
Group × electrode	0.31	0.68	4.18e−5	1.00	9.06e−5
Group × condition	0.31	0.68	0.11	0.88	0.29
Group × electrode × condition	0.05	0.94	3.13e−14	1.00	5.64e−13

## Discussion

Different studies reinforce that sentence comprehension is influenced by various factors, including MSI (Pishghadam et al., 2020, 2021; Shayesteh et al., 2020; Boustani et al., 2021). Given that this type of instruction is believed to activate several regions of the brain, it is considered one of the most effective ways of teaching. In order to explore if this whole-brain instruction technique may further improve learners’ L2 sentence comprehension, if it is followed by two vocabulary repetitions, we adopted a neurocognitive approach and used the ERP method.

The overall behavioral (RA & RT) and electrophysiological findings of the study supported our preliminary hypothesis that, unlike Jajarmi et al.’s (2020) findings, two repetitions may not actually boost the L2 vocabulary knowledge obtained through the MSI. To teach L2 vocabulary items to adults mainly, teachers generally adopt a unisensory approach (audition only), representing what Shayesteh et al. (2019) refer to as *thin education*, or integrate auditory and visual senses (bisensory instruction) and disregard senses of touch, taste, and smell due to limited time and instructional facilities. The influence of vocabulary repetition for this type of learning may not probably be analogous to the one following the MSI. Thus, confirming Nation’s (2001, p. 115) proposition that “the nature of the original learning” determines later retrieval, we suggest that the nature of instruction and learning modulates, to a considerable extent, the effect of subsequent instructional practices – like vocabulary repetition – employed to deepen student learning. Consequently, unlike the studies reporting that learning requires multiple repetitions (e.g., Webb, 2007), we put forward that the (two)repetition effect following multisensory instructional practices seems to be ineffective on L2 sentence comprehension. The justification may probably have its roots in the neurophysiological mechanism underlying the process of learning through multiple senses. The knowledge of words builds up through sensory experiences. That is, different kinds of input that enter the brain through different modalities cumulate and form a comprehensive whole (Shams and Seitz, 2008; Katai, 2011). Throughout this process, greater sensory information gateways and neural networks are activated, and more extended areas of the brain (including sensory-specific and multisensory convergence zones) are engaged (Driver and Noesselt, 2008; Goswami, 2008). Repetition, on the other hand, reinforces the neural connections across the synapses, strengthening the link between form and meaning (Gathercole and Baddeley, 1990), and adds to the quality of knowledge (Nation, 2001), which, in fact, facilitates later retrieval and comprehension. However, given that activating a small proportion of neurons in large scale networks may not affect the network output (Parker, 2010), we assume that the effect of two repetitions, with a short delay period, is so subtle that it does not modify the extensive network of neurons activated through the multiple senses approach.

Holding the view that senses may have a profound impact on vocabulary learning and sentence comprehension, we intend to suggest that, rather than challenging the irrefutable role of repetition as a step forward in language learning, the substantial role of senses in this process needs to be underlined, more than the past, by incorporating sensory-based models, such as emotioncy (Pishghadam et al., 2017; Karami et al., 2019; Makiabadi et al., 2019), into the regular curriculum. Such models lead the old multisensory movement toward entering a new phase which may open up new vistas for teachers and educators. Despite the time-consuming nature of multisensory education, it is strongly recommended that teachers make use of senses in their teaching practices, believing that the considerable merits compensate for the extra efforts exerted by teachers. Another pedagogical implication of the current study could be that two vocabulary repetitions seem redundant after multisensory learning, as opposed to bisensory learning (Jajarmi et al., 2020), since we witnessed no significant change between the cognitive reactions of the two groups. However, raising the number of repetitions may perhaps produce very different results. Therefore, repetition should not be entirely overlooked; instead, it should be more carefully probed and applied.

In the end, there are a few points that need to be taken into careful consideration in future endeavors. First and foremost is that, in addition to the effect of repetition happening in close temporal succession, the effect of spaced repetition with increasingly larger intervals on multisensory vocabulary learning should be meticulously verified since it is believed to produce improved long-term results (Rawson and Kintsch, 2005). Therefore, spacing of the repetitions may allow for probable differences in later performance, generating different results. Moreover, given that the task we used in this study checked the participants’ receptive vocabulary knowledge only, further research is required to investigate the effect of two repetitions on productive vocabulary knowledge as well. Last but not least, as a complementary action, future studies need to compare the cognitive processes underlying repetition after bisensory and MSI. It is needless to say that increasing the sample size may lead to more reliable conclusions.

## Data Availability Statement

The raw data supporting the conclusions of this article will be made available by the authors, without undue reservation.

## Ethics Statement

The studies involving human participants were reviewed and approved by the Ferdowsi University of Mashhad Ethics Committee. The patients/participants provided their written informed consent to participate in this study.

## Author Contributions

RP conceived and designed the experiments. HJ, SS, and AK performed the experiments. HJ and AK analyzed the data. RP, HJ, SS, and AK contributed to reagents, materials, and analysis tools. HJ, SS, and HN wrote the manuscript. RP and HN reviewed and edited the manuscript. All authors contributed to the article and approved the submitted version.

## Conflict of Interest

The authors declare that the research was conducted in the absence of any commercial or financial relationships that could be construed as a potential conflict of interest.

## Publisher’s Note

All claims expressed in this article are solely those of the authors and do not necessarily represent those of their affiliated organizations, or those of the publisher, the editors and the reviewers. Any product that may be evaluated in this article, or claim that may be made by its manufacturer, is not guaranteed or endorsed by the publisher.
